# Designing and Implementing an Assay for the Detection of Rare and Divergent NRPS and PKS Clones in European, Antarctic and Cuban Soils

**DOI:** 10.1371/journal.pone.0138327

**Published:** 2015-09-23

**Authors:** Gregory C. A. Amos, Chiara Borsetto, Paris Laskaris, Martin Krsek, Andrew E. Berry, Kevin K. Newsham, Leo Calvo-Bado, David A. Pearce, Carlos Vallin, Elizabeth M. H. Wellington

**Affiliations:** 1 School of Life Sciences, University of Warwick, Coventry, CV4 7AL, United Kingdom; 2 Ecosystem Programme, British Antarctic Survey, Natural Environment Research Council, High Cross, Cambridge, CB3 OET, United Kingdom; 3 Department of Biomedical Research, Center of Pharmaceutical Chemistry, Atabey, Playa, Havana, Cuba; U. S. Salinity Lab, UNITED STATES

## Abstract

The ever increasing microbial resistome means there is an urgent need for new antibiotics. Metagenomics is an underexploited tool in the field of drug discovery. In this study we aimed to produce a new updated assay for the discovery of biosynthetic gene clusters encoding bioactive secondary metabolites. PCR assays targeting the polyketide synthases (PKS) and non-ribosomal peptide synthetases (NRPS) were developed. A range of European soils were tested for their biosynthetic potential using clone libraries developed from metagenomic DNA. Results revealed a surprising number of NRPS and PKS clones with similarity to rare Actinomycetes. Many of the clones tested were phylogenetically divergent suggesting they were fragments from novel NRPS and PKS gene clusters. Soils did not appear to cluster by location but did represent NRPS and PKS clones of diverse taxonomic origin. Fosmid libraries were constructed from Cuban and Antarctic soil samples; 17 fosmids were positive for NRPS domains suggesting a hit rate of less than 1 in 10 genomes. NRPS hits had low similarities to both rare Actinobacteria and Proteobacteria; they also clustered with known antibiotic producers suggesting they may encode for pathways producing novel bioactive compounds. In conclusion we designed an assay capable of detecting divergent NRPS and PKS gene clusters from the rare biosphere; when tested on soil samples results suggest the majority of NRPS and PKS pathways and hence bioactive metabolites are yet to be discovered.

## Introduction

Emerging multidrug resistant pathogens resistant to nearly all known antibiotics [1], coupled with the ubiquitous spread of antibiotic resistance throughout the wider environment such as in rivers [2], waste water [3] and agriculture [4], has led to an urgent global need for new antibiotics [5]. Natural products have been essential in drug discovery with 60%– 75% of drugs aimed at cancer and infectious disease originating from natural origin [6, 7]. In particular secondary metabolites offer a rich source of bioactive compounds including antibiotics, antifungals, anticancer and immunosuppressants [6]. The two main pathways for production of secondary metabolites consist of the non-ribosomal peptides (NRPs) containing synthetases (NRPSs) and polyketides (PKs) with specific synthases (PKSs) which have contributed to several of the most important human medicines to date such as vancomycin [8], rifamycin [9] and bleomycin [10]. Much of the study and exploitation of secondary metabolites has focused on a culture dependent approach with the advent of genome sequencing revealing a surprising diversity of silent or cryptic gene clusters potentially encoding for a tremendous range of bioactive metabolites [11]. Despite advances in genome mining with several bioinformatics tools allowing for rapid identification of gene clusters [12], comparatively few studies have investigated the use of metagenomics for drug discovery [13]. Metataxonomics has revolutionized microbial ecology [14] with estimates of > 99% of bacteria remaining recalcitrant to culture [15]. Studies of 16S gene sequences using PCR analysis from total community (metagenomic) DNA has led to a greater understanding of the phylogenetic view of bacterial diversity [16]. Targeted study of functional genes through PCR amplification of a marker gene from metagenomic DNA has also been used to look at metabolic diversity of microbial populations such as using *amoA* for analysis of the diversity of ammonia-oxidising communities [17]. Functional metagenomics (whereby genes are captured in plasmid, fosmid or BAC libraries and expressed) has been successfully used to capture and express many functional genes such as those associated with antibiotic resistance [18, 19]. Surprisingly this approach has not been widely adopted for evaluating the diversity of biosynthetic gene clusters. From the limited studies performed in the field of metagenomic drug discovery, several new bioactive compounds have been discovered [20–22]. Indeed a recent study displayed the large metabolic potential of worldwide soils using a PCR assay targeting the pathways involved in synthesis of non-ribosomal peptides and polyketides [23]. Yet the problem when investigating biosynthetic pathway distribution is the assays available for their study. NRPSs and PKSs are modular enzyme complexes producing metabolites in an assembly line fashion by the incorporation of an acyl-CoA or amino acid building block into a growing metabolite [24]. NRPSs are multidomain enzymes consisting of a minimal core structure containing an adenylation (A) domain, condensation (C) domain and peptidepeptidyl carrier protein (PCP). Similarly PKS modules consist of an acyltranferase (AT) domain, ketosynthase (KS) domain and acyl carrier protein (ACP) [24, 25]. The conserved modular nature of NRPS and PKS modules allows for the design of primers on hypervariable regions to analyse the variability across the gene clusters [18, 19]. Much of the work to date performed on biosynthetic gene cluster diversity relies mostly on two PCR assays described over a decade ago [23, 26–28] that are based on higly degenerate primers which may not be beneficial for screening large metagenomic libraries. A possible approach would be to perform shotgun sequencing of all metagenomic DNA, thus removing PCR bias, however a comparison of this method with PCR approaches revealed that the total shotgun metagenomic approach lacked significant depth in comparison with targeted amplicon sequencing [29]. In the current study we aimed to design a new updated PCR assay for NRPS and PKS modules for use in screening metagenomes for biosynthetic pathways. We demonstrate that our PKS and NRPS assays are specific, can detect clusters from a wide range of phyla and have a good hit rate. Here we describe the use of the assay in both prospecting diversity from a range of European soils and screening fosmid libraries from diverse soils. Sites were chosen to represent a range of different habitats to maximise the potential for discovering novel biosynthetic clusters. These included samples from Mars Oasis in Antarctica, which has previously been shown to have a high prevalence of the prolific antibiotic-producing phylum Actinobacteria [30], a high biodiversity site in Cuba proven to be abundant in enzymatic activity [31], and a range of sites from across Europe representing both coastal, untreated hay meadow, and heavily polluted agricultural soil.

## Methods

### Sample sites

Soil samples for the Antarctic fosmid library were taken from Mars Oasis, located on the south eastern coast of Alexander Island on the western Antarctic Peninsula [32]. Cuban samples were taken from the rhizosphere of a sandy location on the Cayo Blanco island as previously described [31]. The European sites from which soil was collected were Druridge Bay, UK (sand dunes), Cockle Park Plot 6, UK (untreated agricultural hay meadow, gleyic brown earth) and the suburbs of Athens, Greece (heavily disturbed agricultural soil). All soils were imported under the Department of Environment, Food and Rural Affairs License No. 51993/1 9493812, For the Antarctic samples the soils were gathered under a Specialist Activities in Antarctica permit issued by the FCO under the Antarctic Act 1994, Antarctic Act 2013 and Antarctic Regulations 1995/490 (as amended). Cuban soils were collected under a collaboration between The School of Life Sciences, University of Warwick and Biotechnology Department, Centre of Pharmaceutical Chemistry, Havana, Cuba. For all other soils no special permits were required.

### Primer design, PCR and sequencing

The NRPS primers were generated from the consensus sequence on the adenylation domain of nine NRPS pathways obtained from GenBank (Table 1) using BLOCKMAKER and CODEHOP [33]. The Type-II PKS primers were generated using the same approach described for NRPS primers aligning 18 KS_α_ genes (Table 2). Reaction mixes were made with 12.5 μl PCR Master Mix (Promega, Madison, WI, USA), 1.25 μl DMSO and 0.8 μM of each primer (Table 3) in 25 μl total volume. The cycling protocol used was the same for all primers with only the annealing temperature varying (Table 3): 5 min denaturing step at 95°C followed by 40 cycles of 30 s at 95°C, 45 s at 61°C or 63°C and 90 s at 72°C followed by a final extension step for 10 min at 72°C. To test the primers, a range of 50 strains with known PKS and NRPS genes were screened (S1 Table). For subsequent screening *S*. *griseus* DSM 40236, *S*. *vinaceus* DSM 40257, *S*. *lividans* 1326 and *S*. *coelicolor* M145 (genomic DNA) were used as positive controls for both primer sets (NRPS_F2/R and PKS_F/R). The PCR products were run on a 1% agarose gel and the product bands were purified using the QIAquick Gel Extraction Kit (QIAGEN; Venlo, Netherlands) as per manufacturer’s instructions. Sequencing was performed using 50 ng of purified PCR product with 5 μM of primer using Sanger sequencing (GATC Biotech AG, Cologne, Germany). Both the forward and reverse primers were used for sequencing to ensure there were no sequencing errors.

**Table 1:** Non-ribosomal peptide synthetases used for primer design.

Accession	Description
gi|2894188|	PCZA363.3 [*Amycolatopsis orientalis*]
gi|4481933|	CDA peptide synthetase II [*Streptomyces coelicolor A3(2)*]
gi|4481934|	CDA peptide synthetase I [*Streptomyces coelicolor A3(2)*]
gi|45006|	Alpha-aminoadipyl-L-cysteinyl-D-valine synthetase [*Amycolatopsis lactamdurans*]
gi|987101|	Pipecolate incorporating enzyme [*Streptomyces rapamycinicus*]
gi|3798625|	GFK506 peptide synthetase [Streptomyces sp. MA6548]
gi|2052277|	Virginiamycin S synthetase [*Streptomyces virginiae*]
gi|2052249|	Pristinamycin I synthase 3 and 4 [*Streptomyces pristinaespiralis*]
gi|5051823|	Putative peptide synthetase [*Amycolatopsis orientalis*]

**Table 2:** Ketoacylsynthases used for primer design.

Accession	Description
gi|125235	KAS1_STRCO Putative polyketide beta-ketoacyl synthase 1 (WhiE ORF III)
gi|729871|	KAS1_STRHA PUTATIVE POLYKETIDE BETA-KETOACYL SYNTHASE 1 (KS) (POLYKETIDE CONDENSING ENZYME)
gi|729870|	KAS1_STRCN PUTATIVE POLYKETIDE BETA-KETOACYL SYNTHASE 1
gi|15823945|	3-oxoacyl-(acyl carrier protein) synthase I [*Streptomyces avermitilis*]
gi|11024335|	PKSA beta-ketoacylsynthase subunit alpha; PKSA-ORF1 [*Streptomyces collinus*]
gi|7209628|	Ketosynthase [*Streptomyces nogalater*]
gi|7209626|	Ketosynthase [*Streptomyces venezuelae*]
gi|2580442|	ORF 1 [*Actinomadura hibisca*]
gi|7433744|	Polyketide synthase *Actinomadura hibisca*
gi|5381247|	Polyketide synthase [*Actinomadura verrucosospora*]
gi|14486277|	B-ketoacyl-ACP synthase-like protein [*Streptomyces aureofaciens*]
gi|125237|	KAS1_STRVN GRANATICIN POLYKETIDE PUTATIVE BETA-KETOACYL SYNTHASE 1
gi|510722|	jadomycin polyketide ketosynthase; JadA [*Streptomyces venezuelae* ATCC 10712]
gi|1076101	ketosynthase–*Streptomyces griseus*
gi|532245|	daunorubicin-doxorubicin polyketide synthase
gi|516109|	polyketide synthase [*Streptomyces*]
gi|7209618|	ketosynthase [*Streptomyces aureofaciens*]
gi|7209610|	ketosynthase [*Streptomyces capoamus*]

**Table 3:** Primers used in this study.

Gene	Primer	Sequence	Amplicon size	Annealing T°C
NRPS	F	CGCGCGCATGTACTGGACNGGNGAYYT	480	63
	R	GGAGTGGCCGCCCARNYBRAARAA		
PKS	F	GGCAACGCCTACCACATGCANGGNYT	350	61
	R	GGTCCGCGGGACGTARTCNARRTC		

A comparison of the new degenerate primer sets with primers already available targeting either the adenylation domain (NRPS) or the ketosynthase domain (PKS) [26–28] was conducted on single strains genomic DNA and on the Cuban metagenomic library. Reaction mixes were made with 12.5 μl PCR Master Mix (Promega, Madison, WI, USA), 1.25 μl DMSO and 0.8 μM of each primer in 25 μl total volume. The following primers and PCR condition were tested: A3F 5’- GCSTACSYSATSTACACSTCSGG-3’ and A7R 5’SASGTCVCCSGTSCGGTAS-3’ [26] (5 min at 95°C followed by 40 cycles of 30s at 95°C, 30s at 59°C, 90s at 72°C and a final step of 5 min at 72°C); degKS2F 5’- GCIATGGAYCCICARCARMGIVT-3’ and degKS2R 5’-GTICCIGTICCRTGISCYTCIAC-3’ (5 min at 94°C followed by 40 cycles of 40s at 94°C, 40s at 55°C, 75s at 72°C and a final step of 5 min at 72°C [27, 28]).

### Screening Antarctic and Cuban fosmid libraries

The construction of the Antarctic fosmid library has been previously described by Pearce *et al*. [30] and the creation of the Cuban fosmid library was performed following the protocol described by Brady [34] using Copy-control^™^ Fosmid Library phage packaging system (Epicentre, Madison, WI, USA). Metagenomic libraries, created in Falcon^®^ 96-well cell culture plates containing LB medium with addition of 12.5 μg/ml chloramphenicol and 1X CopyControl Fosmid Autoinduction Solution (Epicentre), were stored at -80°C and 4°C.

For the screening of both the Antarctic and the Cuban libraries, each 96-well plate was individually pooled using 20 μl from each well and a fosmid extraction was performed using the GeneJET plasmid miniprep kit (Thermo Scientific) as per manufacturer’s instructions. A PCR using 1 μl of the extracted fosmid DNA was performed using the same conditions previously described in order to identify the presence of the target genes. Bands of the expected size were purified from 1% agarose gel using the QIAquick Gel Extraction Kit (QIAGEN; Venlo, Netherlands) and sequenced (GATC Biotech AG, Cologne, Germany) using both forward and reverse primers to avoid sequencing errors. The two libraries of approximately 24,690 and 3000 *E*. *coli* clones containing 30–40 Kb of environmental DNA (eDNA) per fosmid, for a total amount of 864 Mb and 105 Mb respectively for Antarctic and Cuban samples, were screened for NRPS_F/R and PKS_F/R primer sets (Table 1). The Cuban library was also screened with the additional primers A3F/A7R and degKS2F/R following the conditions previously described in order to compare the new primers with the ones already available. All sequenced amplicons can be viewed under GenBank accession numbers KT443010 –KT443022 and KT443093 –KT443096.

### Screening of European soils

Total Community DNA (TCDNA) was extracted from the soils using the CTAB/phenol/chloroform ribolyzing based method [35] and used as a template for PCR with the NRPS_F/R and PKS_F/R primer sets. The resulting PCR products were cloned according to the manufacturer’s instructions (Promega pGEM-T Easy Vector Systems) and 47 NRPS clones and 42 PKS clones were sequenced from each site. The sequences were compared to the GenBank database using the blastn algorithm to confirm that they were of the desired genes and the closest matches in GenBank were included in the analysis. The sequences were aligned using ClustalW in Molecular Evolutionary Genetics Analysis in MEGA [36] and neighbor joining trees were also constructed in MEGA. Bootstraps were preformed with 1000 replicates. All sequenced amplicons can be viewed under GenBank accession numbers KT443023 –KT443092.

## Results and Discussion

### Performance of the primers

Screening of 50 strains as known producers of either PKs or NRPs proved that the primer design detected the majority of an extensive range of genes involved in production of highly diverse antibiotics (S1 Table). The performance of the primers is summarized in S2 Table and revealed that the primer pair NRPS_F/R detected a range (74%) of NRP clusters. The PKS primer pair detected 50% of the strains producing known PKs.

Comparison of the new primers with the existing primers showed that hit rates of the newly designed primers were comparable to other primers with a slightly different distribution of hits (Table 4 and S1 Fig).

**Table 4:** Comparison of primer sets on genomic DNA of different actinomycetes. The positive PCR hits are reported with the + symbol. Examples of known biosynthetic products related to NRPS and PKS clusters present in the strains are reported in the “Antibiotic pathways” column (Source: database ClusterMine360).

	Antibiotic pathways				PCR results			
Organism	NRPS	PKSI	PKSII	Hybrid NRPS-PKS	NRPS_F/R	A3F/A7R	PKS_F/R	degKS2F/R
*Micromonospora fulvoviolaceus* JCM 3258						+		
*Streptomyces avermitilis* MA-4680		+ (Avermectins)			+	+	+	+
*Streptomyces coelicolor* M1152						+	+	
*Streptomyces coelicolor* M1154						+	+	
*Streptomyces coelicolor* M145	+ (CDA)		+ (Actinorhodin)	+ (Prodigiosin)	+	+	+	+
*Streptomyces flavogriseus*							+	
*Streptomyces griseus* DSM 40660								
*Streptomyces hygroscopicus* AM-3672		+ (Herbimycin)			+	+	+	+
*Streptomyces hygroscopicus* NRRL 3602				+ (Geldanamycin)	+	+	+	+
*Streptomyces hygroscopicus subsp*. *glebosus* ATCC 14607								
*Streptomyces lividans* TK24								
*Streptomyces parvulus*		+ (Borrelidin)				+		+
*Streptomyces rochei* DSM 40231	+ (Streptothricin)	+ (Lankamycin)		+ (Lankacidin)	+	+	+	+
*Streptomyces spectabilis*					+	+	+	+
*Streptomyces subrutilus*						+		
*Streptomyces violaceusniger* KCC-S0850				+ (Meridamycin)	+	+	+	+

All the primers sets (NRPS_F/R, A3F/A7R, PKS_F/R and degKS2F/R) were also tested to screen the metagenomic library from Cuban soil to compare any differences in the hit rate and increase the chances of identifying clones with novel antibiotic gene clusters. The screening results showed no positive hits for PKS_F/R and degKS2F/R and an equal number of clones was detected with NRPS_F/R and A3F/A7R. Details of the positive hits is given in Table 5, two clones out of six were positively identified with both primers sets, while the remaining four were identified by a single primers set (two clones with NRPS_F/R and two clones with A3F/A7R).

**Table 5:** Results of nucleotide sequences identity of the positive clones identified during the screening for NRPS and PKS genes of the metagenomic library created from Cuban soil using the blastn algorithm.

**Clone**	**Primers set**	**Species**	**Annotation**	**Accession No.**	**% Identity**	**E value**
ST1P6A4	NRPS_F/R	*Delftia acidovorans* SPH-1	Amino acid adenylation domain protein	CP000884.1	99	0.0
ST1P6A4	A3F/A7R	*Delftia acidovorans* SPH-1	Amino acid adenylation domain protein	CP000884.1	98	0.0
ST1P6B6	NRPS_F/R	*Delftia acidovorans* SPH-1	Amino acid adenylation domain protein	CP000884.1	98	0.0
ST1P6B6	A3F/A7R	*Delftia acidovorans* SPH-1	Amino acid adenylation domain protein	CP000884.1	98	0.0
ST1P9E10	NRPS_F/R	*Saccharothrix espanaensis* DSM 44229	Non-ribosomal peptide synthetase	HE804045.1	80	3e-08
ST1P9D7	A3F/A7R	*Burkholderia gladioli* BSR3 chromosome 2	Arthrofactin synthetase/syringopeptin synthetase C-related non-ribosomal peptide synthetase module	CP002600.1	85	1e-07
ST1P19C8	NRPS_F/R	*Stenotrophomonas maltophilia* R551-3	amino acid adenylation domain protein	CP001111.1	97	2e-174
ST1P29D1	A3F/A7R	*Streptomyces ansochromogenes*	nrps2 metabolite biosynthetic gene cluster	KF170330.1	70	4e-20

### Diversity of PKSs in European soils

**Fig 1:**
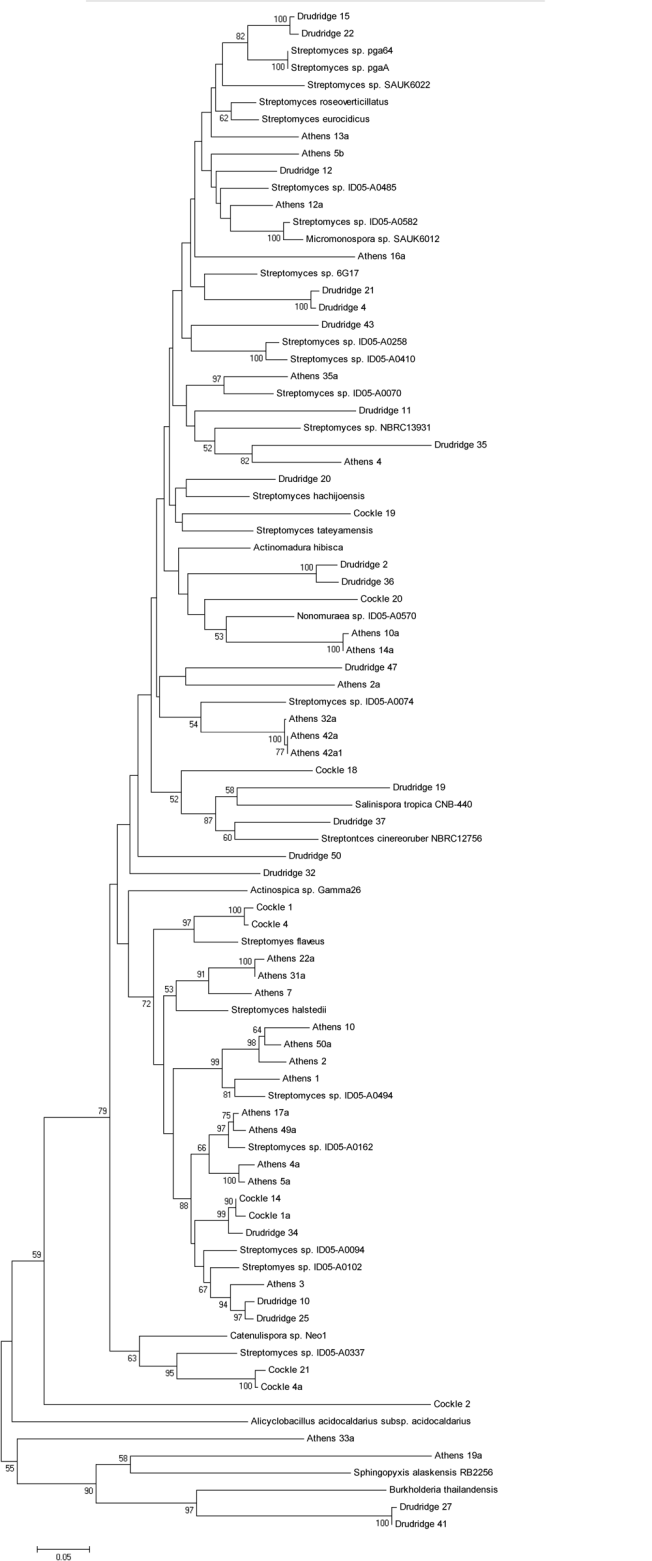
Neighbour joining tree demonstrating relationship between PKS clones recovered from Cockles, Athens and Drudridge. Reference sequences from Genbank were included and are indicated by named species.

PKS primers were developed on conserved regions of 18 different ketosynthase (KS) amino acid sequences. The final PCR product size of 350 bp resulted from two conserved sites flanking a highly variable region allowing for excellent discrimination between gene clusters. Total community DNA was extracted from the Drudridge, Cockles and Athens sites to allow for comparison of the PK biosynthetic gene cluster diversity in the uncultured fraction of the three European soils. PCR products were generated and clone libraries were constructed. For PKS primers 51 products with similarity to KS domains were amplified from the European soils (Fig 1). The KS domains recovered had similarities to a wide range of KS domains present in diverse taxa in the NCBI database. Two major clades were recovered (Fig 1), with a third smaller clade and a few phylogenetically distinct singletons also present. The first major clade included clones recovered with similarity exclusively to the Actinomycetales Order such as *Streptomyces* including *Streptomyces halstei* and *S*. *flaveus*. Other KS domains recovered in this clade had similarity to the rare Suborder Catenulisporineae, including the *Actinospica* and *Catenulispora* genera. The second clade also included clones recovered with similarity to previously described KS domains in the Actinomycetales Order. A large number of clones shared similarity with clones from the genera *Streptomyces*, but different species from clade 1 such as *S*. *cineoruber*, *S*. *hachijoensis*, and *S*. *eurocidius*. However a number of recovered KS domains also shared similarity to rarer actinobacteria such as members of the genus *Nonomuraea* (Athens soils) and the obligate marine actinobacteria *Salinispora tropica* (Druridge). Many clones in the second clade separated having very distant relationships to known PK gene clusters such as Drudridge 32, Drudridge 47 and Athens 2a. Such a distant relationship suggests they are as of yet uncharacterized PKS genes from uncultured actinobacteria. A number of clones were distinct from any of the actinobacteria and although phylogenetically distant, were most similar to KS domains from the Proteobacteria such as *Burkholderia thaliandensis* and *Sphingopyxis alaskensis*. Despite a clear taxonomic spread across the recovered clones, none clustered according to sample site. Clones recovered from each location had representatives in each clade suggesting the PKS gene diversity in this study was not limited by geography. All recovered sequences had an average sequence identity of 71% with a maximum sequence identity of 93% to sequences in the NCBI database, demonstrating the ability of the primers to pick up clusters distinct from those previously observed.

### Testing of NRPS primers on European soils

**Fig 2:**
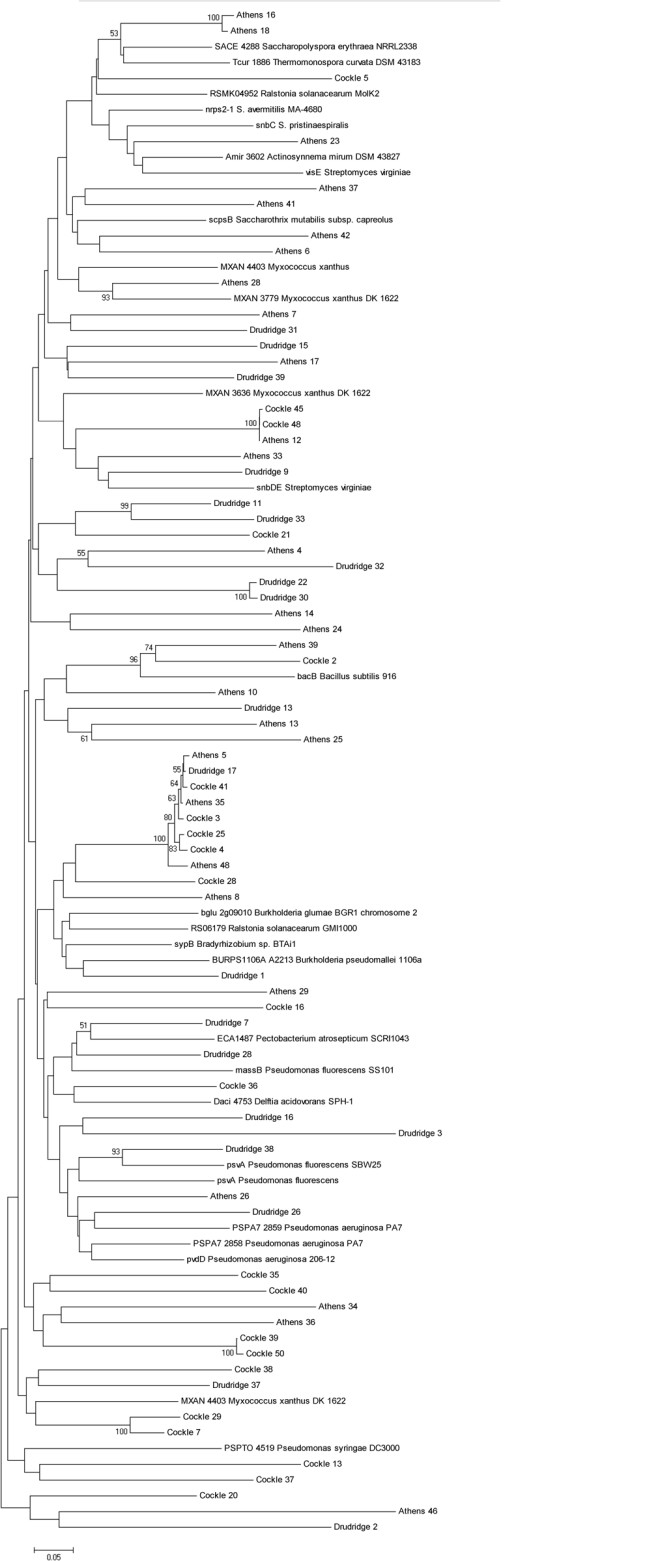
Neighbour joining tree demonstrating relationship between NRPS clones recovered from Cockles, Athens and Drudridge. Reference sequences from Genbank were included and are indicated by named species.

The NRPS_F/R primer set was generated from a consensus sequence of nine NRP pathways and targeted the conserved adenylation (A) domain of the NRPS gene cluster. The final product was 480 bp and flanked a highly variable region allowing discrimination between NRPS biosynthetic genes. Total community DNA extracted from the European soils was used to amplify A domains for the construction of clone libraries to compare the distribution of NRPS gene clusters across samples. A total of 22 clones were amplified from the Cockle Park site, 20 from Drudridge Bay and 28 from Athens (Fig 2). All amplified sequences had similarity to NRPS gene clusters with all blastn hits recorded as ‘peptide synthase’ or ‘amino acid adenylation domain protein’. The sequences recovered clustered with genes originating from a diverse range of bacterial classes including the Alphaproteobacteria (*Bradyrhizobium*), Betaproteobacteria (*Burkholderia*, *Delftia*, *Ralstonia*), Gammaproteobacteria (*Pseudomonas*, *Pectobacterium*), Deltaproteobacteria (*Myxococcus*, *Sorangium*), Bacilli (*Bacillus*) and Actinobacteria (*Actinosynnema*, *Saccharopolyspora*, *Saccharothrix*, *Streptomyces*, *Thermomonospora*). Despite coming from different locations the sequences from the three European soils did not segregate from one another. Several of the sequences had low similarity to any known sequence in the NCBI database, exemplified by Drudridge 2, Athens 46 and Cockle 20. Such clones potentially represent divergent NRPS genes likely to represent biosynthetic gene clusters capable of producing new secondary metabolites.

### Detection of gene clusters from fosmid libraries

**Fig 3:**
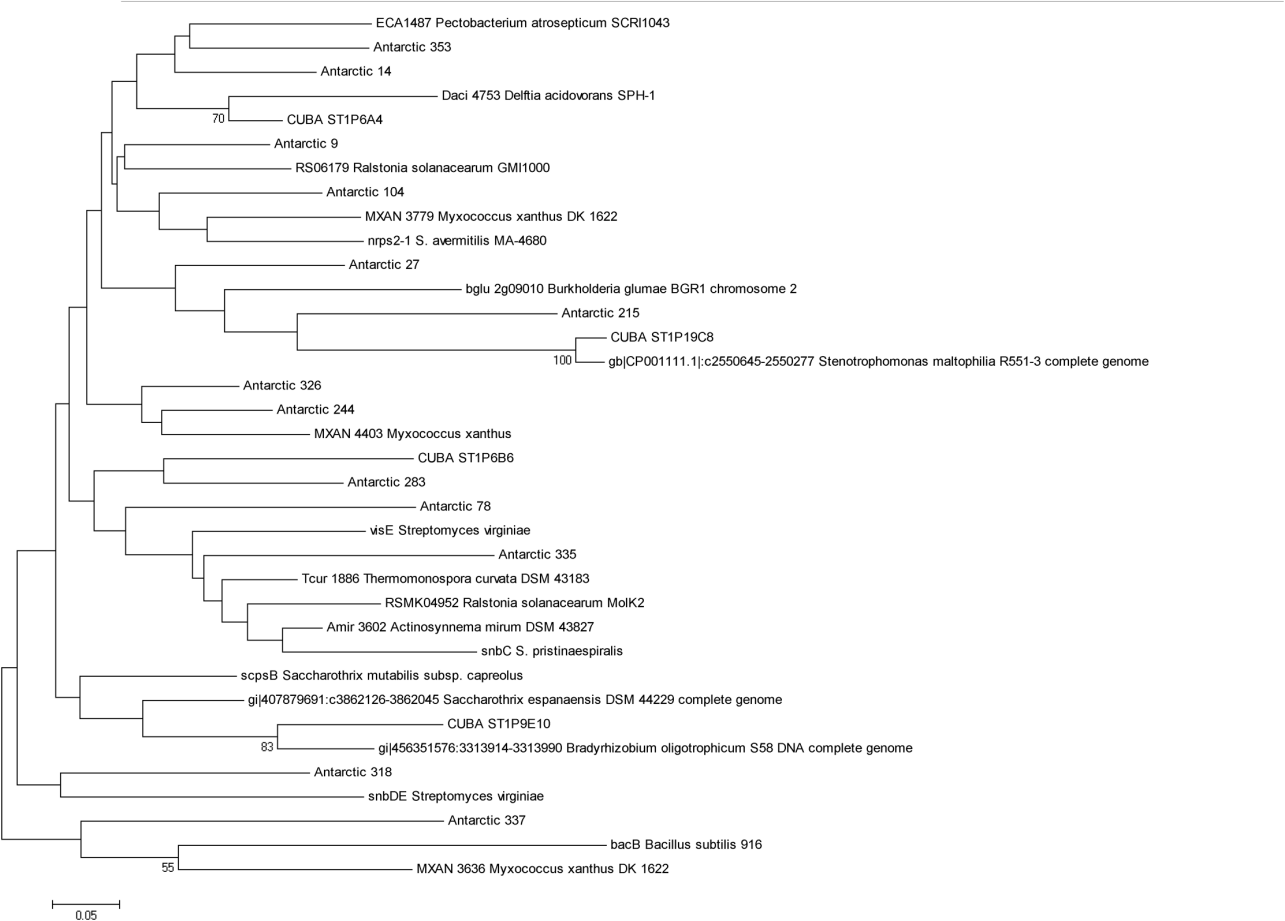
Neighbour joining tree demonstrating relationship between NRPS clones recovered from fosmid libraries constructed from Cuban and Antarctic sample sites. Reference sequences from Genbank were included and are indicated by named species.

In order to analyse the performance of the primer sets in assaying libraries for detection of clusters capable of producing bioactive compounds, pilot fosmid libraries were constructed from soil from both the Antarctic Mars Oasis and Cuban Beach samples. The Antarctic library consisted of ~ 24,690 clones with an average insert size calculated to be approximately 35 kb giving a total library size of 864 Mb. The Cuban library consisted of ~3000 clones with an average insert size calculated to be approximately 35 kb giving a total library size of 105 Mb. Both libraries were screened using the PKS PCR assay (PKS_F/R) and the NRPS PCR assay (NRPS_F/R). A combined total of 17 clones were detected from the two libraries (Fig 3); thus putatively containing a biosynthetic pathway. All hits were recovered using the NRPS PCR assay. From this we can calculate the hit rate in each library as the total DNA captured divided by the number of positive hits (4 in the Cuban library and 13 in the Antarctic library respectively), therefor it is 1 in 26.3 Mb for the Cuban library and 1 in 66.5 Mb for the Antarctic library. Assuming the average *E*. *coli* genome to be 4.6 Mb in size, this suggests that the NRPS assay can detect an average (between the two libraries) greater than one gene cluster per 10 genomes. Although collectively the Antarctic and Cuban NRPS clones had similarity to genes reported in a wide range of phyla, Cuban clones primarily had similarity to sequences found in gram-negative bacteria, specifically the Proteobacteria. In contrast the Antarctic clones had similarities to sequences found in both Proteobacteria as well as a wide range of Actinobacteria including the genera *Thermomonospora*, *Saccharothrix* and *Streptomyces*. Several of the clones had low similarities to sequences of NRPS genes in the NCBI database and were phylogenetically divergent from representative sequences suggesting many of the clones recovered came from as yet undiscovered NRP pathways.

## Discussion

In comparison to genome mining, relatively few studies to date have taken advantage of metagenomics as a tool for drug discovery [13], and those that have had great success in discovering new compounds [21, 22]. To facilitate future drug discovery the aims of this study were to provide additional PCR assays for the capture of biosynthetic pathways, test the biosynthetic potential of different types of soils and demonstrate the assays’ utilities in screening fosmid libraries. We demonstrate here two highly specific assays; all PKS clones had highest similarity to either keto-synthase (KS) or β-ketoacyl ACP synthases from PK-like gene clusters, and all NRPS clone and library hits matched adenylation (A) domains from NRP gene clusters. When screening European soils the PKS assay was able to detect clones from a range of Actinobacteria and Proteobacteria, suggesting these are the most two dominant phyla producing polyketides in the soils tested. This reflects what has previously been observed in culture [37]. Surprisingly many of the clones with similarity to sequences in Actinobacteria were similar to PK pathways from rare genera such as *Actinospica*, *Catenulispora* and *Nonomuraea*. All three of the genera have been recorded to produce potent antimicrobials such as the chrolactomycins from *Actinospica* [38], novel aminocoumarins from *Catenulispora* [39] and a novel drug targeting *Mycobacterium tuberculosis* ecumicin from *Nonomuraea* [40]. As well as rare actinobacteria, clones were similar to sequences from the classic natural product producing *Streptomyces* [11]. Similar findings were reported for the NRPS assay, with recovered clones having similarity to sequences from a diverse range of bacterial classes belonging to the Actinobacteria and Proteobacteria phyla, such as the Delta Proteobacterium *Myxococcus*, a prolific antibiotic producer [41]. The ability of the assays to amplify clones from such a diverse range of taxa is indicative of the flexibility of both described assays. Many of the functional genes amplified from clones recovered from European soils were phylogenetically divergent from representatives in the NCBI database suggesting they were from novel gene clusters, which demonstrates the ability of the assays to detect new NRPS and PKS pathways. This also infers that European soils may have a wide range of untapped bioactive potential as has been described for soil in general [23]. Despite previous studies suggesting biosynthetic pathways may be restricted by biogeography [23], here we did not observe this for either PKS or NRPS gene sequences, although the number of clones was low and a greater sequencing effort is needed to discover all the gene clusters in these soils. The amplicon size of 350 bp for PKS and 480 bp for the NRPS are both compatible with next generation sequencing, allowing for greater sequencing depth in future studies. The primers worked well for the recovery of clones in fosmid libraries, indicating the presence of biosynthetic gene clusters containing NRPS and PKS genes; the future characterization of the recovered fosmids may lead to the discovery of interesting bioactive compounds. The Cuban site appeared to contain a greater diversity of target sequences with similarity to Proteobacteria which correlates well with a previous community analysis proving prevalence of alpha-proteobacteria in this soil [31].

In conclusion, in this study we have validated two new assays for drug discovery targeting the PKS and NRPS genes involved in the biosynthesis of many antibiotics. The two assays were capable of bioprospecting new environments and mining fosmid libraries. They are a useful addition to the current selection of primers used for bioprospecting metabolic diversity in environmental samples and extend the range of gene clusters detected. The results support the hypothesis that a range of Actinobacteria and Proteobacteria are responsible for producing diverse PKs and NRPs. Assays were capable of detecting novel diverged variants of previously described NRPS and PKS gene clusters, and detected sequences found in phylogenetically distinct groups, which implies a lack of bias. Fosmid libraries constructed from soils recovered a number of clones with a high hit rate for clusters of genes potentially capable of producing bioactive compounds, supporting the research of diverse soils in drug discovery.

## Supporting Information

S1 Fig Comparison through PCR amplification of all primers on genomic DNA from actinobacteria. PCR amplicons obtained with primers: A) PKS_F/R, B) degKS2F/R, C) NRPS_F/R, D) A3F/A7R. The numbers represent the following strains: 1) *S*. *griseus* DSM 40660, 2) *S*. *hygroscopicus* AM-3672, 3) *S*. *violaceusniger* KCC-S0850, 4) *S*. *subrutilus* 445, 5) *S*. *hygroscopicus supsp*. *glebosus* ATCC 14607, 6) *S*. *coelicolor* M145, 7) *S*. *coelicolor* M1154, 8) *S*. *coelicolor* M1152, 9) *S*. *lividans* TK24, 10) *S*. *avermitilis* MA-4680, 11) *S*. *rochei* DSM 40231, 12) *S*. *flavogriseus*, 13) *Micronomospora fulvoviolacea* JCM 3258, 14) *S*. *specatibilis*, 15) *S*. *parvulus* 1038, 16) *S*. *hygroscopicus* AM-3602.
(TIF)

S1 Table Summary of primer testing against a set of 50 reference strains. (DOCX)

S2 Table A summary of primer testing results. (DOCX)
