# Supplementary figures and images for: Designing and Implementing an Assay for the Detection of Rare and Divergent NRPS and PKS Clones in European, Antarctic and Cuban Soils
Source: PLoS One. 2015 Sep 23;10(9):e0138327. doi: 10.1371/journal.pone.0138327 (PMC4580463; doi:10.1371/journal.pone.0138327)

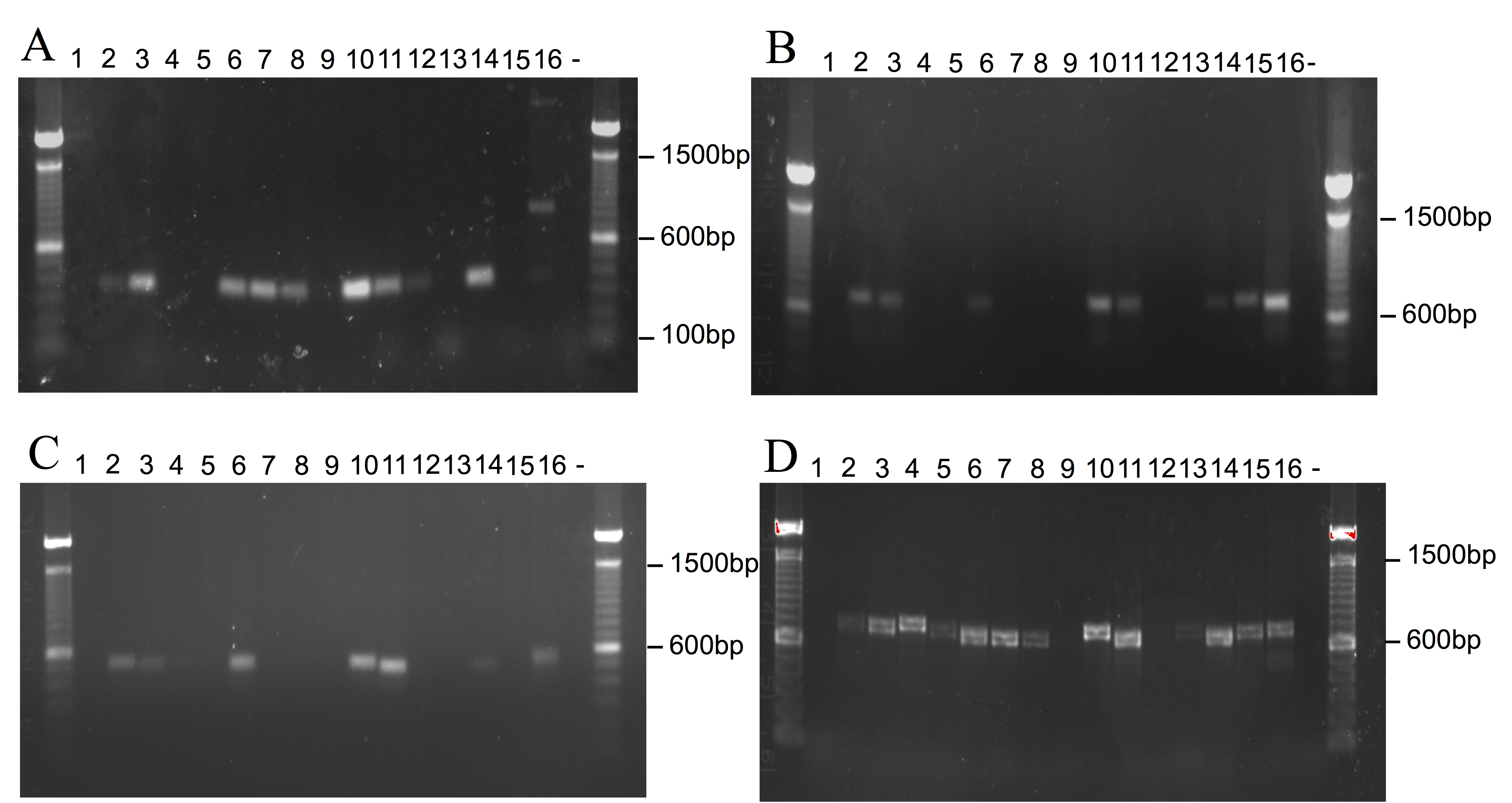

Supplement: S1 Fig — PCR amplicons obtained with primers: A) PKS_F/R, B) degKS2F/R, C) NRPS_F/R, D) A3F/A7R. The numbers represent the following strains: 1) S. griseus DSM 40660, 2) S. hygroscopicus AM-3672, 3) S. violaceusniger KCC-S0850, 4) S. subrutilus 445, 5) S. hygroscopicus supsp. glebosus ATCC 14607, 6) S. coelicolor M145, 7) S. coelicolor M1154, 8) S. coelicolor M1152, 9) S. lividans TK24, 10) S. avermitilis MA-4680, 11) S. rochei DSM 40231, 12) S. flavogriseus, 13) Micronomospora fulvoviolacea JCM 3258, 14) S. specatibilis, 15) S. parvulus 1038, 16) S. hygroscopicus AM-3602. (TIF) [file pone.0138327.s001.tif]
