# Supplementary material for: Designing and Implementing an Assay for the Detection of Rare and Divergent NRPS and PKS Clones in European, Antarctic and Cuban Soils
Source: PLoS One. 2015 Sep 23;10(9):e0138327. doi: 10.1371/journal.pone.0138327 (PMC4580463; doi:10.1371/journal.pone.0138327)
Supplement: S1 Table — (DOCX) [file pone.0138327.s002.docx]

**S1 Table** Summary of primer testing against a set of 50 reference strains; + and – represent the presence and absence of an amplicon respectively. Examples of known antibiotic biosynthetic products related to NRPS and PKS clusters present in the strains are reported in the “Biosynthesis” column with related pathway described in the adjacent column.

| **Strain** | **Biosynthesis** | **Pathway** | **NRPS_F2/R** | **PKS_F/R** |
| --- | --- | --- | --- | --- |
| *Actinomadura kijaniata* ATCC31588 |  |  | **-** | **-** |
| *Amycolatopsis azurea* ATCC 51273 | Glycopeptide antibiotic | NRPS | **+** | **-** |
| *Amycolatopsis lactamdurans* ATCC 27382 | Cephalosporin antibiotic | NRPS | **+** | **+** |
| *Amycolatopsis mediterranei* ATCC 13685 | Ansamycin-type antibiotic | PKS I | **+** | **+** |
| *Amycolatopsis orientalis ssp. orientalis* ATCC 19795 | Glycopeptide antibiotic | NRPS | **+** | **-** |
| *Amycolatopsis sulphurea* ATCC 27624 | Antibiotic related to the tetracyclines | PKS II | **+** | **+** |
| *Amycolatopsis alba* ATCC 51368 | Glycopeptide antibiotic | NRPS | **-** | **-** |
| *Amycolatopsis fastidiosa* ATCC 31181 |  |  | **+** | **-** |
| *Micromonospora olivasterospora* ATCC 21819 |  |  | **+** | **+** |
| *Nocardia uniformis* JCM 3224 | beta-Lactam antibiotic | NRPS | **+** | **+** |
| *Nonomuraea roseoviolacea ssp. carminata* DSM 44170 | Anthracycline antibiotic | PKS II | **-** | **+** |
| *Streptomyces michiganensis* ATCC 14970 | Depsipeptide antibiotic | NRPS | **+** | **-** |
| *Streptomyces chrestomyceticus* ATCC 14947 |  |  | **+** | **-** |
| *Streptomyces eurythermus* ATCC 14975 | Macrolide antibiotic | PKS I | **+** | **-** |
| *Streptomyces azureus* ATCC 14921 | Peptide Antibiotic | NRPS | **+** | **+** |
| *Streptomyces coeruleorubidus* ATCC13470 | Anthracycline antibiotic | PKS II | **+** | **-** |
| *Streptomyces collinus* ATCC 19742 | Antibiotic |  | **+** | **+** |
| *Streptomyces neyagawaensis* ATCC 27449 | Macrolide antibiotic | PKS I | **+** | **+** |
| *Streptomyces resistomycificus* ATCC 19804 | Quinone-related antibiotic | PKS II | **+** | **+** |
| *Streptosporangium vulgare* ATCC 33329 | Macrolide antibiotic | PKS I | **+** | **+** |
| *Streptosporangium album* DSM 43023 |  |  | **-** | **-** |
| *Streptomyces olivaceus* (Warwick 44) |  | PKS II | **+** | **+** |
| *Streptomyces olivaceus* (Warwick 48) | Anthracycline antibiotic | PKS II | **+** | **-** |
| *Streptomyces sp.* (Warwick 53) |  | PKS II | **+** | **+** |
| *Streptomyces antibioticus* (Warwick 56) | Angucycline antibiotic | PKS I, PKS II | **-** | **-** |
| *Streptomyces sp.* (Warwick 57) | Macrolide antibiotic complex | PKS I | **+** | **-** |
| *Streptomyces capreolus* NRRL 3817 |  |  | **-** | **-** |

^♯^Abbreviations: ATCC (American Type Culture Collection); DSM (Deutsche Sammlung von Mikroorganismen und Zellculturen GmbH); JCM (Japan Collection of Microorganisms); NRRL (Agricultural Research Service Culture Collection); Warwick (University of Warwick culture collection).

**S1 Table** Cont.

| **Strain** | **Biosynthesis** | **Pathway** | **NRPS_F2/R** | **PKS_F/R** |
| --- | --- | --- | --- | --- |
| *Saccharotrix australiensis* ATCC 31497 |  |  | **+** | **-** |
| *Streptoallotheicus hindustanus* ATCC 31217 |  |  | **-** | **-** |
| *Streptomyces ghoshikiensis* ATCC 23914 | Macrolide-type antibiotic | PKS I | **+** | **+** |
| *Streptomyces bottropensis* ATCC 25435 | Cyclic peptide antibiotic complex | NRPS | **-** | **-** |
| *Streptomyces macrosporeus* ATCC 21388 | Polypeptide antibiotic complex | NRPS | **+** | **-** |
| *Streptomyces paulus* NRRL 12251 |  | NRPS | **+** | **-** |
| *Nocardia asteroids* ATCC 9969 |  | NRPS | **+** | **+** |
| *Actinoplanes serveparensis* (Warwick 102) |  |  | **+** | **-** |
| *Streptomyces vinaceus* ATCC 11861 | Cyclic peptide antibiotic | NRPS | **+** | **+** |
| *Streptomyces afghaniensis* ATCC 23871 |  | PKS II | **+** | **+** |
| *Streptomyces achromogenes ssp. achromogenes* JCM 4121 |  |  | **-** | **+** |
| *Streptomyces citricolor* JCM 5028 |  |  | **+** | **+** |
| *Streptomyces durhamensis* JCM 4747 | Pentaene antibiotic complex | PKS I | **+** | **+** |
| *Streptomyces fellus* ATCC 19752 | Macrolide antibiotic | PKS I | **+** | **-** |
| *Streptomyces griseus ssp. griseus* JCM 5012 |  | NRPS | **+** | **-** |
| *Streptomyces humidus ssp. humidus* JCM 4386 |  |  | **+** | **+** |
| *Streptomyces limosus* ATCC 19788 |  |  | **+** | **-** |
| *Streptomyces lincolnensis* JCM 4287 |  |  | **-** | **+** |
| *Streptomyces rishirensis* JCM 4821 |  |  | **-** | **+** |
| *Streptomyces tauricus* JCM 4837 | Anthracycline antibiotic complex | PKS II | **+** | **+** |
| *Streptomyces tuirus* ATCC 19007 | Antibiotic |  | **-** | **-** |
| *Streptomyces violaceoruber* JCM 4979 | Triketide | PKS | **-** | **+** |
| *Streptomyces sparsogenes* JCM 4517 |  |  | **+** | **-** |

^♯^Abbreviations: ATCC (American Type Culture Collection); DSM (Deutsche Sammlung von Mikroorganismen und Zellculturen GmbH); JCM (Japan Collection of Microorganisms); NRRL (Agricultural Research Service Culture Collection); Warwick (University of Warwick culture collection).
