# Supplementary material for: Designing and Implementing an Assay for the Detection of Rare and Divergent NRPS and PKS Clones in European, Antarctic and Cuban Soils
Source: PLoS One. 2015 Sep 23;10(9):e0138327. doi: 10.1371/journal.pone.0138327 (PMC4580463; doi:10.1371/journal.pone.0138327)
Supplement: S2 Table — (DOCX) [file pone.0138327.s003.docx]

**S2 Table** A summary of primer testing results.

| **Primer set** | **Positives (%) of total** | **Positives that correlate with metabolic information*/ (%) of total** | **Product not observed when expected.** |
| --- | --- | --- | --- |
| NRPS_F2/R | 74 | 22 | 2 |
| PKS_F/R | 50 | 24 | 12 |
